# PUFKEY: A High-Security and High-Throughput Hardware True Random Number Generator for Sensor Networks

**DOI:** 10.3390/s151026251

**Published:** 2015-10-16

**Authors:** Dongfang Li, Zhaojun Lu, Xuecheng Zou, Zhenglin Liu

**Affiliations:** School of Optical and Electronic Information, Huazhong University of Science and Technology, No.1037 Luoyu Road, Wuhan 430074, China; E-Mais: lidongfang@hust.edu.cn (D.L.); d201377521@hust.edu.cn (Z.L.); estxczou@gmail.com (X.Z.)

**Keywords:** RNG, PUF, high security, high throughput

## Abstract

Random number generators (RNG) play an important role in many sensor network systems and applications, such as those requiring secure and robust communications. In this paper, we develop a high-security and high-throughput hardware true random number generator, called PUFKEY, which consists of two kinds of physical unclonable function (PUF) elements. Combined with a conditioning algorithm, true random seeds are extracted from the noise on the start-up pattern of SRAM memories. These true random seeds contain full entropy. Then, the true random seeds are used as the input for a non-deterministic hardware RNG to generate a stream of true random bits with a throughput as high as 803 Mbps. The experimental results show that the bitstream generated by the proposed PUFKEY can pass all standard national institute of standards and technology (NIST) randomness tests and is resilient to a wide range of security attacks.

## 1. Introduction

In recent decades, sensor networks have been becoming a widely-used technology [1,2,3]. As is well known, those networks are made up of a large number of small nodes that sense the environment and typically report their measurements to a base station. Because of the need for the protection against unauthorized access, the trustworthiness of transmitted messages and data integrity in general, security issues are very important and cannot be neglected when developing sensor network applications.

Commonly-implemented security mechanisms rely on the availability of random numbers in order to perform their operations, as in the case of key exchange algorithms [4], which are based on randomly-generated keys, or of mutual authentication algorithms [5], which use the so-called random “nonce” to ensure the other part is trusted. Random number generators therefore play a crucial role when considering security in sensor networks.

Currently, two basic methods can be used to generate random numbers.

The first method is a true random number generator (TRNG). It requires a physical source, which is truly random and from which bits can be derived directly. Such non-deterministic sources derive their randomness from underlying properties that exhibit unpredictable behavior. Recently, there have been some great TRNGs. Pyo, Pae and Lee [6] presented a DRAM-based TRNG, which exploits the unpredictability in DRAM access time caused by refresh cycles. Wieczorek [7,8] designed a novel TRNG that exploits random behavior from a nearly-metastable operation of groups of FPGA flip-flops as opposed to many deep metastability-based TRNGs. Amaki, Hashimoto and Onoye [9] presented an oscillator-based TRNG that automatically adjusts the duty cycle of a fast oscillator to 50% and generates unbiased random numbers tolerating process variation and dynamic temperature fluctuation. There are two important downsides to most of these TRNG constructions. Firstly, some require specific hardware to extract the randomness from the physical entities on the device [10]. Secondly, the throughput of TRNGs is generally relatively low [11]. They are problematic when large streams of random bits are required for cryptographic applications in sensor networks.

The second approach to generate a random number is by using true random seeds and pseudo-random number generators (PRNGs), which are deterministic algorithms. Some of them use SRAM PUF or other kinds of PUF to generate a secure random seed, then take advantage of PRNGs to generate a random bitstream [12,13,14,15]. It can produce a stream of random output bits at a high throughput rate. However, The output of such a generator only seems random to observers without prior knowledge. If an observer knows which data have been used as a seed for the PRNG or some output of a random number, then he/she will be able to calculate all output values of the generator.

In this paper, we propose PUFKEY, a high-security and high-throughput hardware true RNG, to resolve the above problems. We use SRAM PUF to generate a high quality random seed, which is non-deterministic. Subsequently this initial seed is fed into another stable PUF that generates a larger amount of random bits. The random bitstream is unpredictable. At the same time, it could generate a random bitstream at a high rate with tight cost, area and power constraints. More importantly, it can resist a wide range of security attacks.

The remainder of this paper is organized as follows. Section 2 provides related work in PUF and a SRAM cell. We present the architecture of PUFKEY in Section 3. After that, we analyze and evaluate entropy in start-up values in Section 4, a conditioning algorithm in Section 5 and non-deterministic random number generator (NDRNG) in Section 6. Based on these analyses, we discuss the security of PUFKEY in Section 8 after the implementation in Section 7. Finally, we conclude the paper in Section 9.

## 2. Related Work

### 2.1. PUF

PUFs are most commonly used for the identification or authentication of integrated circuits (ICs), either by using the inherent unique device fingerprint or by using it to derive a device unique cryptographic key [16]. Due to deep submicron manufacturing process variations, every transistor in an IC has slightly different physical properties that lead to measurable differences in terms of its electronic properties, such as the threshold voltage, gain factor, *etc.* Since these process variations are uncontrollable during manufacturing, the physical properties of a device can neither be copied nor cloned. It is very hard, expensive and economically inviable to purposely create a device with a given electronic fingerprint. Therefore, one can use this inherent physical fingerprint to uniquely identify an IC.

### 2.2. SRAM as Sources of Entropy

**Figure 1:**
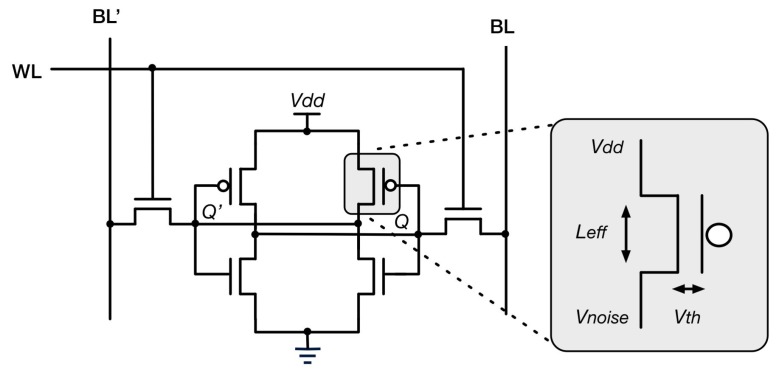
SRAM cell with relevant process variation and noise shown.

Our approach to generating a seed value is based on random noise extracted from the power-up state of SRAM modules. Each bit of SRAM is a six-transistor storage cell consisting of cross-coupled inverters and access transistors. Each of the inverters drives one of the two state nodes labeled as A and B. When the circuit is unpowered, both state nodes are low (QQ’ = 00). Once power is applied, this unstable state will immediately transition to one of the two stable states, which are either “0” (QQ’ = 01) or “1” (QQ’ = 10). The choice between the two stable states depends on threshold mismatch and thermal and shot noise. Because the stabilization depends only on mismatch between local devices, the impact of common mode process variations, such as lithography, and common mode noise, such as substrate temperature and supply fluctuations, is minimized. The sources of randomness are shown in Figure 1.

Therefore, uninitialized SRAM is normally considered to be in a logically-unknown state. Some cells, however, will be almost perfectly symmetric, which leads them to settle to an unpredictable value at start-up. It is the noise due to these cells that we exploit in order to generate high quality random seeds.

## 3. Architecture

**Figure 2:**
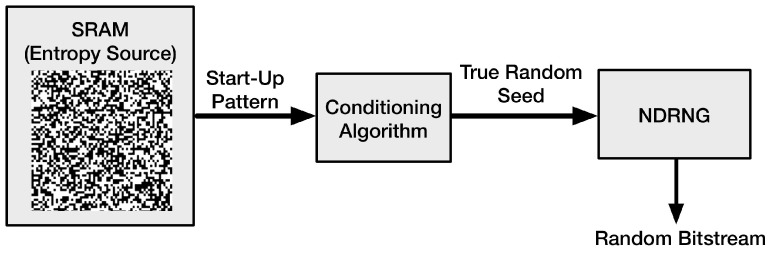
The architecture of PUFKEY.

In this section, we propose the architecture of PUFKEY, which is depicted in Figure 2. PUFKEY consists of two main parts:

(1) An SRAM memory connected to a conditioning algorithm for deriving a truly random seed.

(2) A non-deterministic random number generator (NDRNG).

Because of the design of an SRAM memory, a large part of the bit cells is skewed due to process variations and tends to start up with a certain preferred value. This results in a device-unique pattern that is present in the SRAM memory each time it is powered. Bit cells that are more symmetrical in terms of their internal electrical properties, on the other hand, tend to sometimes start up with a “1” value and sometimes with a “0” value. Hence, these bit cells show noisy behavior. This noise can be used for the generation of random bitstreams.

The conditioning algorithm is used to derive a truly random seed from the SRAM start-up values. As explained above, only part of the SRAM bits have noisy behavior when being powered. The entropy in these noisy bits needs to be condensed into a full entropy random seed. The conditioning algorithm takes care of this. Basically, the conditioning algorithm is a compression function that compresses a certain amount of input data into a smaller fixed size bit string. The amount of compression required for generating a full entropy true random output string is determined by the min-entropy of the input bits.

Finally, the generated hash is used to seed a non-deterministic random number generator, which can generate a stream of true random numbers. It is a vital part in the whole system, because it is at the core of contributing to the high throughput and high security.

## 4. Evaluation of Entropy in SRAM Start-Up Values

For the purpose of extracting a random seed from SRAM start-up values, it is important to investigate and analyze their entropy contents. In this section, we explain the approach to quantify the entropy quality of SRAM patterns. To make sure that this seed is truly random, the SRAM patterns should be conditioned (e.g., hashed, compressed, *etc.*). This conditioning will be discussed in the next section.

### 4.1. Method of Deriving Min-Entropy

In order to extract a high quality seed from the SRAM start-up values, we have to examine their randomness properties in terms of entropy. In particular, the amount of entropy that must be present in the noise of SRAM start-up patterns should be determined. Therefore, we will calculate the min-entropy. This method is based on the NISTspecification [17] that defines min-entropy as the worst case (*i.e.*, the greatest lower bound) measure of uncertainty for a random variable.

For a binary source, we can define the min-entropy as:

(1) $$H_{min}=-log_{2}\left(max\left(p_{0},p_{1}\right)\right)$$

In Equation (1), $p_{0}$ and $p_{1}$ are the probabilities of “0” and “1” occurring. Assuming that all bits from the SRAM start-up pattern are independent, each bit i can be viewed as an individual binary source. For each of these sources, we estimate the probabilities $p_{0}^{i}$ and $p_{1}^{i}$ of powering up “0” or “1” respectively by repeatedly measuring the power-up values of the SRAM. In case *m* subsequent measurements are performed, $p_{0}^{i}$ denotes the number of occurrences of a zero divided by m and $p_{1}^{i}$ = 1 – $p_{0}^{i}$. For n independent sources (where n is the length of the start-up pattern), we have:

(2) $$H_{min}=\sum_{i=1}^{n}-log_{2}\left(max\left(p_{0}^{i},p_{1}^{i}\right)\right)$$

Hence, under the assumption that all bits are independent, we can sum the entropy of each individual bit to derive the min-entropy of the entire SRAM. In the remainder of this work, we generally denote the available min-entropy as a percentage of the total available SRAM size.

### 4.2. Measurement of Entropy

To be able to calculate the min-entropy of the noise on SRAM memories under different environmental conditions, measurements are performed on SRAM-based FPGA. Since it is known that SRAM start-up patterns are susceptible to external influences (such as deep-submicron process variations, varying ambient temperature, voltage variations, *etc.*), it is important to measure the min-entropy under different circumstances.

Cortez [18] has researched the modeling and analysis of their start-up values (SUVs) of SRAM. He presents an analytical model for SUVs of an SRAM PUF based on the static noise margin (SNM) and reports some industrial measurements to validate the model. The model is further used to perform a sensitivity analysis to identify the impact of different technology and non-technology parameters. Simulation of the impact of different sensitivity parameters (such as variation in power supply, temperature, transistor geometry) has been performed. The results show that out of all sensitivity parameters, variation in threshold voltage is the one with the highest impact in technology parameters. Schrijen [19] concludes that temperature is a key influencing factor in non-technology parameters. Supply voltage variation does not influence the reliability of these SRAM memories when used as PUFs. Selimis [20] also proves that the temperature is an important element. Therefore, we choose to perform measurements at varying ambient temperatures in our work.

For min-entropy calculations, the measurement environment for the worst case behavior should be as stable as possible. Therefore, we will be determining the minimal amount of entropy for each of the individual ambient temperature conditions.

We measured the entropy on 10 samples. The samples are from block SelectRAM (18Kb) on the Xilinx Virtex-II Pro Platform, which contains a Xilinx XC2VP30 [21]. We calculated the min-entropy for all of these memories at −10 °C, +20 °C and +50 °C. Using these measurements, the results for the different conditions can be found in Table 1.

**Table 1:** Min-entropy results at different temperatures (Min-entropy denoted as the percentage of total available block RAM).

Device	$-10^{{}^{\circ}}C$	$+20^{{}^{\circ}}C$	$+50^{{}^{\circ}}C$
Board 101	4.1%	5.1%	5.8%
Board 102	4.3%	5.3%	5.7%
Board 103	4.3%	5.3%	6.0%
Board 104	4.0%	5.2%	5.9%
Board 105	4.2%	5.1%	6.1%
Board 106	4.5%	5.0%	5.9%
Board 107	4.3%	5.4%	5.8%
Board 108	4.2%	5.4%	5.8%
Board 109	4.0%	5.1%	6.0%
Board 110	4.4%	5.2%	6.3%

### 4.3. Discussion of Measurement Result

From the results in the previous subsection, it becomes clear that all studied memories have different amounts of randomness that can be extracted from noise. In general, we can say that the entropy of the noise is minimal at the lowest measured temperature of −10 °C.

Based on the results, we can conclude that for each of the evaluated memories, it should be possible to derive a truly random seed (with full entropy) of 128 bits from a limited amount of SRAM. For instance, if we assume a conditioning algorithm that compresses its input to 4% (each studied memory has a min-entropy that is higher than 4% under all circumstances), the required length of the SRAM start-up pattern is 400 bytes.

## 5. Conditioning Algorithm

As described in the prior section, a seed is required before generating true random output bits with NDRNG. This seed is used to instantiate NDRNG and to determine its initial internal state. According to the specifications by NIST [17], the seed should have entropy that is equal to or greater than the security strength of the NDRNG. In our design, the NDRNG requires an input seed of 128 bits with the full entropy. In order to achieve this, a conditioning algorithm can be used.

To extract a truly random seed from the SRAM start-up noise, we have selected a lightweight hash function which is called QUARK [22] to perform the conditioning. QUARK could provide at least 64-bit security against all attacks (collisions, multi-collisions, distinguishers, *etc.*), fit in 1379 gate-equivalents, and consume, on average, 2.44 μW at 100 kHz in 0.18 μm ASIC.

This hash function compresses input strings into an output string at the length of 128 bits. In order to have the full entropy, the amount of entropy at the input of the hash function should be at least 128 bits. For example, if the input string has a min-entropy of 4%, the length of this input needs to be at least 400 bytes. Then, we perform the Hamming distance test to find out whether the seed derived by the conditioning algorithm is truly random. For this test, subsets of the generated seeds are compared to each other based on fractional Hamming distance (HD), which is defined by:

(3) $$HD\left(\bar{x},\bar{y}\right)=\sum_{i=1}^{n}x_{i}⊕y_{i}$$

(4) $$Fractional\;HD\left(\bar{x},\bar{y}\right)=\frac{\sum_{i=1}^{n}x_{i}⊕y_{i}}{n}$$

**Figure 3:**
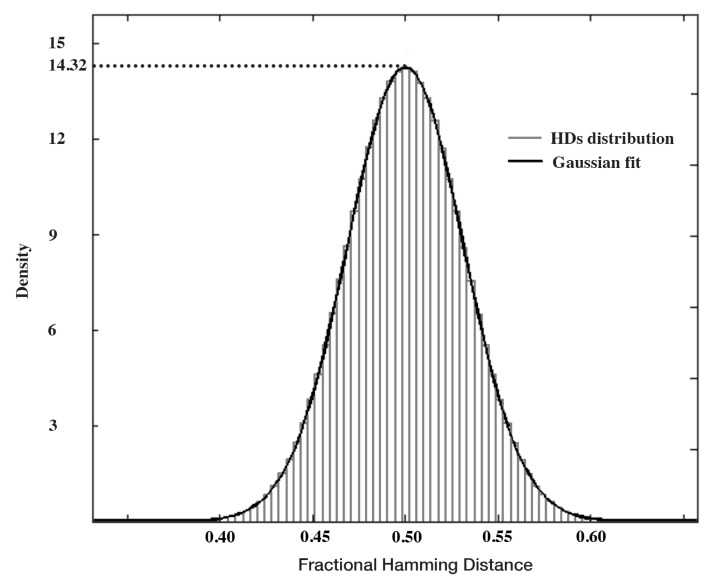
Hamming distance distribution.

Based on Equation (4), $\bar{x}$ and $\bar{y}$ are SRAM start-up patterns with individual bits $x_{i}$ and $y_{i}$, respectively. A set of fractional HDs is composed. HDs are measured 5000 times, and the average value of HDs is 14.327 HDs. To indicate that these seeds have been created by a random source, the set of HDs should have a Gaussian distribution with mean value of 0.5 and a small standard deviation. Figure 3 shows the Hamming distance distribution of the 5000 seeds generated by the conditioning algorithm. As can be seen in this figure, the distribution is perfectly Gaussian with a mean value μ of 0.5. The standard deviation σ of this distribution is 0.04419. The estimation of the entropy of this dataset can be evaluated:

(5) $$\mathrm{Entropy}=\frac{\mu *(1-\mu )}{\sigma^{2}}=\frac{0.5^{2}}{0.04419^{2}}=128\mathrm{bits}$$

Based on this evaluation of 5000 seeds, it appears that the output strings of the conditioning algorithm are truly random and contain the full entropy, since the length of these strings is 128 bits.

## 6. NDRNG

**Figure 4:**
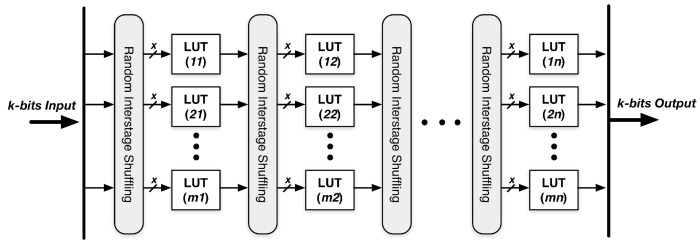
Non-deterministic random number generator (NDRNG).

When we have a truly random seed with full entropy, a stream of random numbers could be generated by NDRNG. The architecture of NDRNG is depicted in Figure 4. It has k bits input/output and nlevels of LUTs. Each LUT has *x* inputs and one output, and it randomly chooses each input from the output of an LUT in the previous levels. The randomness of NDRNG is based on the randomness of inter-stage shuffling and the content of each LUT.

A k-bits random number is generated after using the seed (k-bits) for initialization. The generated k-bits random number would serve as the seed for the next round. We repeat the procedure to continuously generate k parallel random bits at a time and then transform k parallel bits into a sequential bitstream.

**Figure 5:**
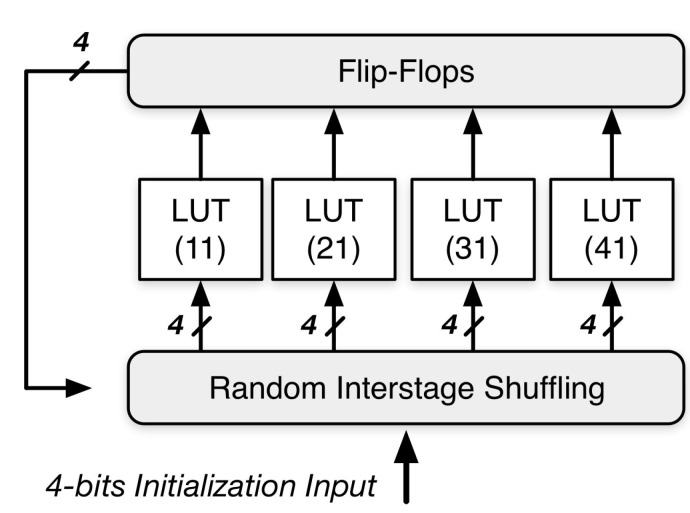
A simple example of NDRNG.

Figure 5 shows a simple example, which has four inputs, four LUTs and four flip flops. The structure is a sequential logic cluster. This design forms a cycle, which can keep repeating. When it comes to the first cycle, a random seed of four bits is used as the primary inputs. The four LUTs select their inputs from the four bits of initialization inputs after random shuffling. Each LUT randomly chooses four bits as its inputs. Then, in Cycle 2, the four outputs generated by the four LUTs in Cycle 1 are stored in the flip flops, and they are becoming the cycle’s inputs after random shuffling. The cycles can be repeated many times.

We choose the Virtex-II Pro Platform to build the architecture of NDRNG. After its power-up, SRAM cells will be altered to either a stable “1” or “0”. Therefore, the inter-stage shuffling and content of SRAM cells in the LUT is randomness. Alternatively, customers can allocate LUTs in such a way that each cell has the same probability to be either “1” or “0”, so the number of “0” and “1” will be completely the same.

**Figure 6:**
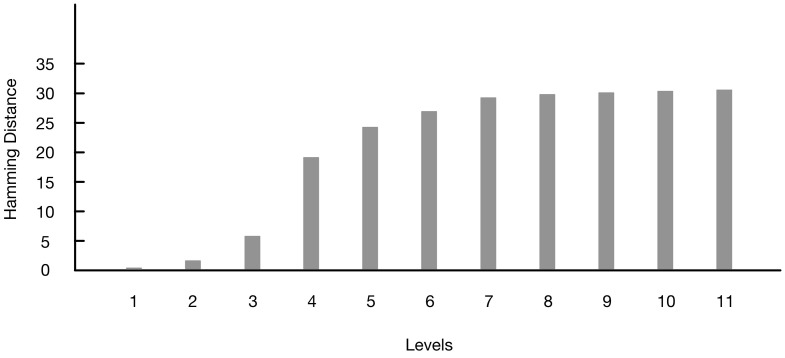
Hamming distance distribution.

An important problem is what the sufficient number of LUT levels is to achieve the good security property while maintaining a low area/power consumption given an NDRNG of k outputs. Given an NDRNG of 128 primary inputs and 128 final outputs and each level of k = 128 input LUTs, we test the output Hamming distance of the avalanche effect for different numbers of levels. The output Hamming distance is defined as the number of bits changed in the output vector when one bit is changed in the input vector. Figure 6 clearly shows that the Hamming distance grows exponentially in the beginning (levels < 5) when the number of levels increases and quickly converges to the ideal case of 128 when the number of levels reaches nine. Therefore, a total of nine levels is enough to achieve a good security property.

The throughput of PUFKEY mainly depends on NDRNG, and we have tested the throughput of NDRNG when the bit width input/output = 128. The results are shown in Section 7.

## 7. Implementation

Based on the above results and analysis, we implemented the proposed RNG in this paper, which consists of SRAM, the conditioning algorithm and NDRNG. The building blocks are combined in the implementation as depicted in Figure 2.

The amount of SRAM used for the implementation is 400 bytes, which are based on the min-entropy estimations from Section 4. The minimal amount of observed min-entropy in this section is 4%. As stated in Section 5, the conditioning algorithm used in this implementation is the u-Quarkhash function, which is the smallest version of Quark. u-Quark has a maximum parallelism degree of eight, meaning that up to eight rounds can be performed within one cycle. Aumasson [22] specifies 4b = 544 rounds. However, further studies show that 3b or even 2b rounds are enough. Therefore, we use the 3b-round permutation of u-Quark. With 3b = 408 rounds, this translates to 408/8 = 51 cycles to process eight bytes. To process 400 bytes, it needs about 2550 cycles.

The random seed is used as the input for NDRNG, which converts the seed into a stream of random bits. The implementation of NDRNG sets m = 128 and n = 9.

The implementation targets the XUP Virtex-II Pro Development System from Digilent. A comparison with related works is summarized in Table 2. A design with multiple ring oscillators is proposed in [23]. However, the underlying assumptions behind the proof of security have been challenged. In addition, the design consumes too much FPGA resources, because it requires 110 ring oscillators. A compact TRNG with a security proof implemented on the Xilinx Spartan-3E FPGA is presented in [24], but this design achieves a throughput of only 250 kb/s. The design based on self-timed ring-oscillators is presented in [25]. This design achieves 100 Mb/s throughput, and it is accompanied by an entropy model, but it consumes too much resource on the FPGA, since it requires a self-timed ring oscillator with 511 stages. A novel entropy extraction technique for true random number generators on FPGAs is presented in [26]. This technique relies on the carry-logic primitives for efficient sampling of the accumulated jitter. However, the highest throughput is 14.3 Mb/s. There is another TRNG comprised of logic gates only [27], and it can be integrated in any kind of logic large scale integration (LSI), but the entropy source determines the maximum data flow (bit rate) of a generator and its sensitivity to the supply voltage.

**Table 2:** Comparison of related work.

Work	Platform	Resources(Slices)	Throughout(Mbps)
Schellekens [20]	Virtex-II Pro	565	2.5
Cherkaoui [21]	Cyclone 3	>255	133
Cherkaoui [21]	Virtex-5	>255	100
Varchola [22]	Spartan 3E	Not reported	0.25
Rozic [23]	Spartan 6	67	14.3
Rozic [23]	Spartan 6	40	1.53
Hisashi [24]	Virtex-4	580	12.5
This work	Virtex-II Pro	369	803

Our design achieves higher throughput than other TRNGs. The maximum delay of NDRNG is 159.22 ns. It is suitable in sensor networks where large streams of true random bits are required for cryptographic applications. However, it costs some hardware resource.

## 8. Security Analysis

### 8.1. Output Randomness

We test the output randomness by applying the NIST randomness test. NIST is a battery of standard statistical tests to detect non-randomness in the binary sequences constructed. Table 3 shows the average passing ratio of each NIST statistical test. One thousand bitstreams of 1,000,000 bits are provided to each test. Each test passes for *p*-value ≥ δ, where δ = 0.01. We can see that the proportion of successful tests is high enough to indicate excellent randomness in the output stream.

**Table 3:** Result of NIST randomness test.

StatisticalTest	Result
Frequency	99.7%
Block Frequency (m = 128)	99.0%
Cusum-Foward	98.4%
Cusum-Reverse	98.5%
Runs	96.9%
Longest Runs of Ones	97.7%
Rank	99.3%
Spectral DFT	97.2%
Non-overlapping Templates (m = 9)	96.0%
Overlapping Templates (m = 9)	96.9%
Universal	99.7%
Approximate Entropy (m = 8)	97.8%
Random Excursions (x = +1)	98.9%
Random Excursions Variant (x = –1)	97.6%
Serial (m = 16)	99.2%
Linear Complexity (M = 500)	97.8%

### 8.2. Security of the Truly Random Seed

What is crucial for security is to maintain the unpredictability of the data stream produced by PUFKEY. Once an attacker knows the seed, it may affect the security of the whole system. Thus, to ensure high security, it is important to protect the seed value from the accesses of other algorithms.

In this work, we assume an attack scenario in which an adversary has no direct physical access to the FPGA. Otherwise, it would be impossible to ensure that the power-up SRAM value remains secret, since an adversary can use a debugging interface, such as JTAG, to halt the FPGA during start-up, read out the data and then let the start-up process continue.

To limit the exposure of the initial SRAM state and prevent attacks where the seed is re-calculated from the SRAM content, all SRAM (except for the seed value) should be cleared immediately after seed generation. This can be achieved by making sure that the seeding algorithm is the very first code that runs on power-up and that the algorithm is executed atomically. Methods to ensure this, such as disabling interrupts and preventing unauthorized firmware modifications, are outside the scope of this paper.

Finally, in order to guarantee proper SRAM resets in between power cycles of the FPGA, care should be taken that the FPGA’s positive supply lines are grounded when the device shuts down. If this is not done, it might power up with old and predictable data of low entropy still being present in SRAM.

### 8.3. Output Hamming Distance

While using the NDRNG to secure the sensor network, the resistance against malicious attack/prediction is important. Even if attackers get seeds or some outputs of random numbers, it is still difficult to calculate the bitstream without the knowledge of NDRNG. In this section, we statistically analyze the security of the NDRNG system. The attacks would be regarded as successful if the output stream can be predicted. The simulation is conducted on an NDRNG architecture with *m* = 128 and *n* = 9. The statistical results are based on 1,000,000 input-output pairs.

**Figure 7:**
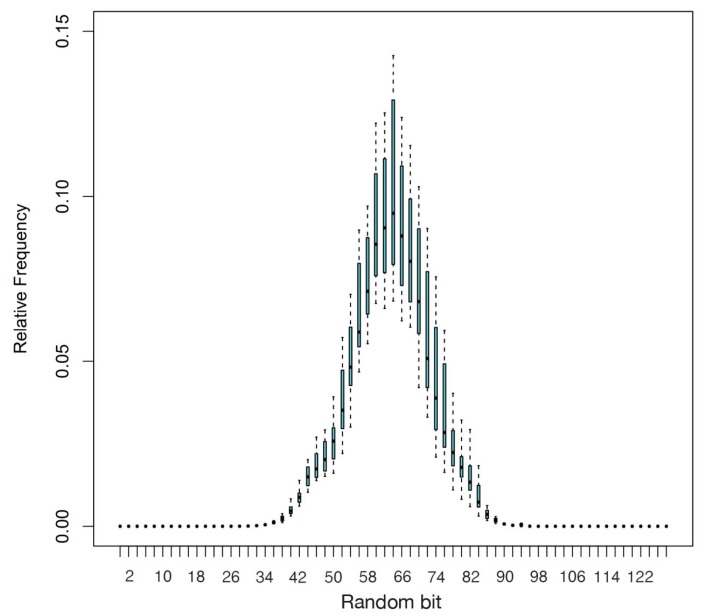
Hamming distance distribution.

One type of prediction is to predict outputs by the knowledge of the outputs from similar inputs. This attack is dangerous when the output vectors with similar input vectors are highly similar to each other. To test this, we summarize the Hamming distances (the number of bits that are different between two vectors) between the output vectors by changing one bit of the input vectors at each iteration. In the ideal case, the distribution would be in the form of a binomial distribution with the peak on the half of the number of outputs. Figure 7 shows the accumulative results of 1000 test cases, and in each case, 1,000,000 instances of the Hamming distance are tested. In the test, we individually tested the 128 output ports of NDRNG. Each output of NDRNG is presented as the X-axis and relative frequency as the Y-axis. The binomial distribution proves that the NDRNG output stream cannot be predicted in this way.

### 8.4. Input-Output Correlation

**Figure 8:**
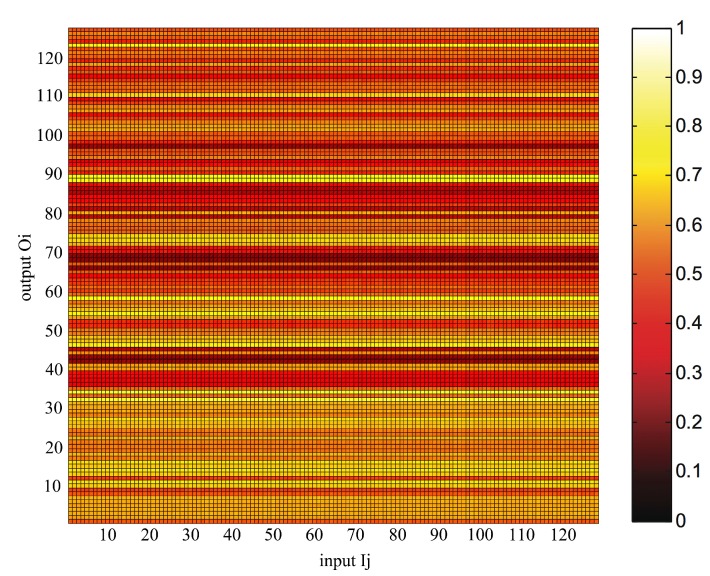
The conditional probability map of P($O_{i}$ = 1|$I_{j}$ = 1).

Similar to the previously-described attack, the other type of attack attempts to predict the output bit $O_{i}$ according to the value of an input bit $I_{j}$ in NDRNG. In either case, if the output bit has a strong correlation with an input bit, then the attacker can deduce the output vector by knowing the input bits. This security test reveals the bitwise correlation between the inputs and the outputs of NDRNG. The 128 output ports of NDRNG are tested individually. We present a conditional probability map of P($O_{i}$ = 1|$I_{j}$ = 1) in Figure 8 depicting the low potential for prediction based on input to output correlation in NDRNG.

### 8.5. Frequency Prediction

**Figure 9:**
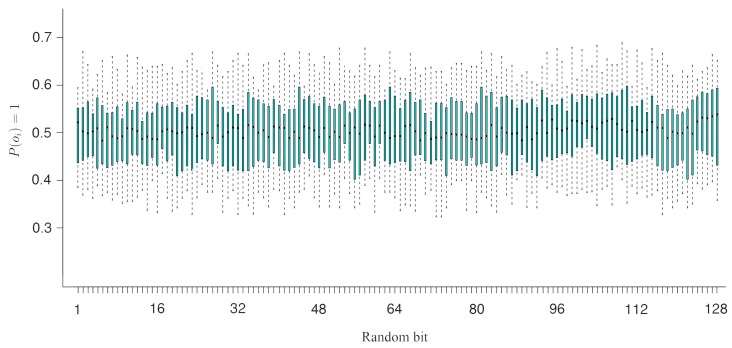
Probability that an output bit is equal to “1”.

There is another attack. The attacker collects the output data from the previous bitstream and builds a probability distribution for each output. Ultimately, the attacker tries to predict each output bit by using this distribution. The goal of the attacker is to predict $P(o_{i})=x$ where *x* = “0” or “1”. The ideal situation is that an output is “0” or “1”, and each output has a probability 0.5. Figure 9 shows the mean value of the probability that each output bit is equal to “1”. In the test, we individually tested the 128 output ports of NDRNG. Each output port of NDRNG is presented as the X-axis and the frequency as the Y-axis. The probability is close to 0.5, indicating the high unpredictability of the structure. We further adopt von Neumann correction to the output of the random bitstream, so that the “0” and “1” have the same probability of 0.5.

## 9. Conclusions

In this work, we present a novel hardware true random number generator PUFKEY for sensor networks, which is based on the combination of extracting a non-deterministic true random seed from the noise on the start-up pattern of SRAM memories and a non-deterministic random number generator to convert this seed into a stream of true random bits. The extraction of the physical randomness from SRAM start-up patterns is based on min-entropy calculation. Then, we use a lightweight hash function QUARK as a conditioning algorithm to extract truly random seeds from SRAM noise. The results show that the seeds contain full entropy. Combining the extracted randomness with the NDRNG, PUFKEY could generate a true random bitstream at 803 Mbps. PUFKEY passes all standard NIST randomness test and resists a wide range of security attacks.
